# Reversible Self-Assembly of Backbone-Thermoresponsive Long Chain Hyperbranched Poly(*N*-Isopropyl Acrylamide)

**DOI:** 10.3390/polym8020033

**Published:** 2016-01-28

**Authors:** Ting-Ting Liu, Wei Tian, Yan-Li Song, Yang Bai, Peng-Li Wei, Hao Yao, Hong-Xia Yan

**Affiliations:** 1 The Key Laboratory of Space Applied Physics and Chemistry, Ministry of Education and Shanxi Key Laboratory of Macromolecular Science and Technology, School of Science, Northwestern Polytechnical University, Xi’an 710072, China; ntt1997@126.com (T.-T.L.); songyanli0514@163.com (Y.-L.S.); yuehe130@126.com (P.-L.W.); yaohao19910901@126.com (H.Y.); hongxiayan@nwpu.edu.cn (H.-X.Y.); 2 Xi’an Mordern Chemistry Research Institute, Xi’an 710065, China; wanan213@126.com

**Keywords:** hyperbranched polymer, reversible self-assembly, poly(*N*-isopropyl acrylamide), thermo-responsiveness, controlled release of drug

## Abstract

In this paper, we mainly described the reversible self-assembly of a backbone-thermoresponsive, long-chain, hyperbranched poly(*N*-isopropyl acrylamide) (LCHBPNIPAM) in aqueous solution. Here, we revealed a reversible self-assembly behavior of LCHBPNIPAM aqueous solution derived from temperature. By controlling the temperature of LCHBPNIPAM aqueous solution, we tune the morphology of the LCHBPNIPAM self-assemblies. When the solution temperature increased from the room temperature to the lower critical solution temperature of PNIPAM segments, LCHBPNIPAM self-assembled from multi-compartment vesicles into solid micelles. The morphology of LCHBPNIPAM self-assemblies changed from solid micelles to multi-compartment vesicles again when the temperature decreased back to the room temperature. The size presented, at first, an increase, and then a decrease, tendency in the heating-cooling process. The above thermally-triggered self-assembly behavior of LCHBPNIPAM aqueous solution was investigated by dynamic/static light scattering, transmission electron microscopy, atomic force microscopy, fluorescence spectroscopy, ^1^H nuclear magnetic resonance in D_2_O, and attenuated total reflectance Fourier transform infrared spectroscopy. These results indicated that LCHBPNIPAM aqueous solution presents a reversible self-assembly process. The controlled release behaviors of doxorubicin from the vesicles and micelles formed by LCHBPNIPAM further proved the feasibility of these self-assemblies as the stimulus-responsive drug delivery system.

## 1. Introduction

Within the last decades, there has been increasing research interest in the various hyperbranched polymers because of their unique chemical and physical properties, such as highly-branched architectures with low viscosity and good solubility, as well as plenty of terminal groups for chemical modifications [1,2,3,4]. Particularly, connecting long, linear polymer chains into a hyperbranched structure leads to some unique properties in comparison with traditional hyperbranched polymers, for instance, good mechanical properties in bulk and responsive properties of temperature, pH, and so on. Thus, long-chain hyperbranched polymers (LCHBPs) have potential applications in various fields, such as drug carrier, energy storage, nanotechnology, and catalysis [5,6].

Up to now, much attention has been paid to the synthesis and properties of LCHBPs. For the synthesis, many step-growth and chain-growth approaches have been invented, which produce a series of LCHBPs such as polystyrene (PSt) [7,8,9,10], polyphenylen/es [11] polysiloxanes [12], polyethers [13,14] and so on. The major strategy for the synthesis of LCHBPs is the polycondensation of AB_n_ (*n* ≥ 2) macromonomers [15,16,17]. For example, Hutchings *et al.* reported that long-chain hyperbranched PSt was synthesized by anionic polymerization of a well-defined linear AB_2_ type PSt macromonomer [7,10,18]. Alternatively, the click reaction of alkyne with azide has been proved to be highly efficient [19,20] and has been extensively applied in the synthesis and modification of various polymer materials [21,22,23]. Pan *et al.* first utilized alkynyl-azide click reaction to make well-defined hyperbranched PSt by atom transfer radical polymerization (ATRP) of linear AB_2_ type PSt macromonomer containing an azide group at its one end and two terminal propargyl groups at the other end [24]. Wu *et al.* [9,15,25,26] obtained a series of long seesaw-type hyperbranched PSt by the click reaction between azide and alkynyl groups of linear AB_2_ seesaw-type PSt macromonomers which have one alkynyl group in the middle and one azide group at each chain end. For another aspect, studies on the structure-property relationships of LCHBPs have been established [27,28,29,30,31,32,33,34]. It was found that long chain hyperbranched poly(l-lactide)s and polyimides have a significant impact on the rheological properties of such materials in the melt [33].

Until now, however, there are not enough researches that focus on the self-assembly of LCHBPs. Although long chain hyperbranched poly(ε-benzyloxycarbonyl-l-lysine) conjugated with thiol-terminated poly(ethylene oxide) could self-assemble into nearly solid micelles in aqueous solution [35], this kind of micelles is hardly able to respond to external stimulus. In another example, Wu *et al.* [36] studied the thermoresponsive behavior of star-like copolymers with long chain hyperbranched PSt as the core and poly(*N*-isopropyl acrylamide) (PNIPAM) as the grafting chains in aqueous solution. However, the hyperbranched backbone of this star-like copolymer was not responsive to external stimuli and the deeper self-assembly behavior has not been systematically investigated. Furthermore, there are few studies on the reversible self-assembly of LCHBPs. Generally, polymers that undergo a reversible transition between water-soluble and water-insoluble states have been particularly attractive and intensively investigated in recent years, since such a reversible transition generally does not require additional chemical reagents to induce the switch [37]. Therefore, our aim in this study is to regulate the reversible self-assembly process of backbone-thermoresponsive LCHBP in aqueous solution.

**Scheme 1:**
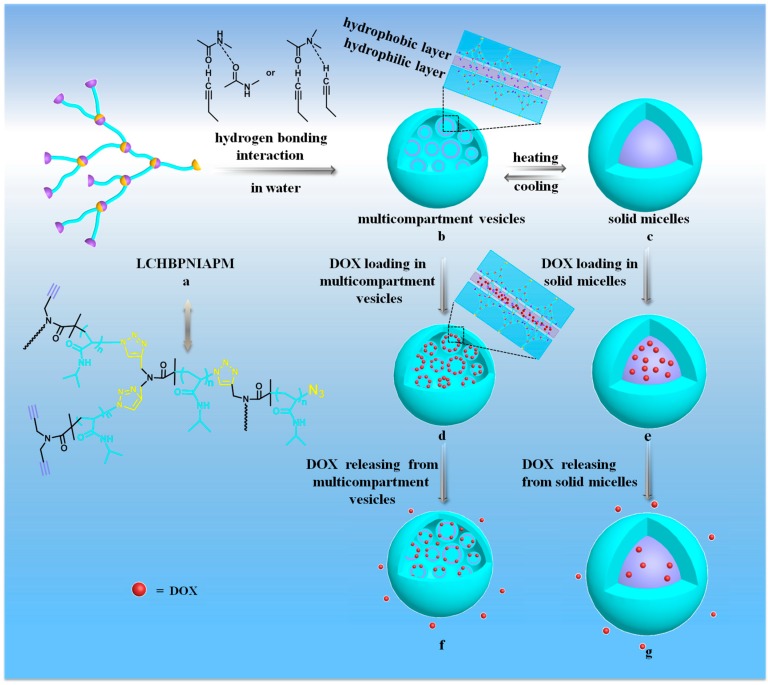
Schematic representation for the possible self-assembly mechanism of LCHBPNIAPM in aqueous solution at 20 °C (**a**,**b**) and in the reversible heating-cooling process (**b**,**c**). The release behaviors of DOX from different LCHBPNIAPM self-assemblies; (**b**–**f**) DOX loading in, and releasing from, multi-compartment vesicles at 20 °C; (**c**–**g**) DOX loading and releasing from solid micelles at 37 °C.

In this work, the hyperbranched polymer with long-chain PNIPAM backbone (LCHBPNIPAM) was first synthesized according to our recent work [38]. The thermo-induced reversible self-assembly behavior of LCHBPNIPAM in aqueous solution was then investigated. In the heating-cooling process, the morphology of LCHBPNIPAM self-assemblies can be reversibly regulated from solid multi-compartment vesicles to solid micelles, and back to initial multi-compartment vesicles (Scheme 1a–c). Controlled release results of Doxorubicin (DOX) from LCHBPNIPAM self-assemblies, including multi-compartment vesicles (Scheme 1b–f) and solid micelles (Scheme 1c–g), demonstrated the potential application in the biomedical field.

## 2. Materials

Tris[2-(dimethylamino)ethyl]amine (Me6TREN, 99%, Alfa Aesar, Shanghai, China), and NIPAM (99%, Acros, Shanghai, China) were used as received. *N*,*N*,*N′*,*N*″,*N*″-pentamethyldiethylenetriamine (PMDETA) was supplied by Yutian Chemical, Ltd. (Liyang, China) and used as received without further purification. 4-Dimethylaminopyridine (DMAP, 95%) was purchased from Sinopharm Chemical Reagent Co., Ltd., Shanghai, China. 8-Anilino-1-naphthalenesulfonic acid ammonium salt hydrate (ANS) and was purchased from Alfa Aesar China. Doxorubicin hydrochloride (DOX·HCl, 99%) was purchased from Sigma. CuBr was stirred with acetic acid overnight, then washed with ethanol and dried under vacuum at 25 °C. Other reagents were purchased from Tianjin Kermel Chemical Reagents Development Center (Tianjin, China). They were dried with 4 Å-grade molecular sieves before use without further purification. The synthesis of LCHBPNIPAM can be seen in supporting information.

## 3. Characterization Methods

### 3.1. Polymer Solution Characterization

The size and morphology of the micelles with different polymer concentrations 0.02 mg/mL were revealed by TEM (Hitachi H-7650, Tokyo, Japan) at an acceleration voltage of 80 kV. The samples were prepared according to the literature [39,40,41,42]. The polymer solutions were treated at given temperatures for an hour, and 10 μL of the self-assembled aggregate solution was then sprayed on a carbon-coated TEM grid (300 mesh). The grid, along with the solution sample, was frozen by placing into liquid nitrogen. The spraying and freezing operation was accomplished in *ca.* 2 s. The frozen samples were subsequently freeze dried by slow warming to room temperature. The morphology was visualized using an AFM with a tapping mode and a Nanowizard II controller (Benyuan, CSPM 5500, Guangzhou, China). Tip information: radius ≤ 33 mm, cantilever length 10 μm; width 100 μm; thickness 30 mm, resonant frequency 300 kHz, force constant 40 N/m. A Zetasizer Nano-ZS DLS (Malvern Instruments, Malvern Worcestershire, UK) was used to determine the hydrodynamic diameter of self-assemblies. Each sample was kept at a predetermined temperature for 3 min before measurement without any filter. SLS analysis was performed on a DAWN HELEOS-II multi-angle light scattering detector (Wyatt Technology Corporation, Santa Barbara, CA, USA) operated at 665 nm, using gallium-arsenic as the incident laser beam source. SLS data were collected at six different concentrations of the aggregates and 18 different angles for each concentration. The data were analyzed using the Zimm plot method on HELEOS-II Firmware 2.4.0.4 advanced software to determine *R_g_*. The emission spectra were recorded by FL (Hitachi F-4600, Tokyo, Japan) from 355 to 550 nm with an excitation wavelength at 335 nm. The existence of hydrogen bonding in the self-assembly process of the LCHBPs solutions were processed by FTIR-ATR spectra. A solution of the sample in either THF or water was placed in the liquid cell, and the spectra of the solutions were recorded.

### 3.2. Polymer Solution Properties

The LCST of LCHBPNIPAM was determined by Zetasizer Nano-ZS DLS (Malvern Instruments, Malvern Worcestershire, UK). The *D_z_* value of LCHBPNIAPM aqueous solutions with a constant polymer concentration of 0.2 mg/mL were recorded under different temperature conditions. Sample cells were thermostated with an internal constant temperature controller. The temperature ramp was set at 1 °C/min. The LCST values of the LCHBPNIPAM were defined as the suddenly increasing of *D_z_* value during the heating process.

The guest encapsulation of LCHBPNIPAM was measured by FL (Hitachi F-4600, Tokyo, Japan) using ANS (0.05 mM) as guest molecule in a buffer solution with ionic strength equal to 0.1 mol/L. Typically, the LCHBPNIPAM solution was diluted step-by-step to various desired concentrations (from 0.2 to 2.0 mg/mL) using different guest molecule solutions. All solutions were maintained for more than 12 h to ensure the binding equilibrium and then stirred prior to measurement.

### 3.3. Drug Loading and Vitro Release

The encapsulation and controlled release experiments of DOX were shown as follows. LCHBPNIPAM (20.0 mg) was dissolved in DMF (2.0 mL) and stirred for 2 h. Then DOX·HCl (6.0 mg) was dissolved in mixture solutions. Then 15 μL triethylamine was added dropwise to the solution and the mixture was stirred 12 h to reach equilibration. The unloaded free drug and the salt produced by neutralization reaction were removed by dialysis using a dialysis tube (cut off *M*_n_ 8000–14,000) against 1000 mL pure water at 20 °C with 300 r/min of stirring. Pure water was renewed for six times within 12 h (every 2 h). The final drug loading polymer aqueous had been lyophilized for use. For the release of DOX, the DOX-loaded polymers were dissolved in 5 mL of phosphate-buffered solutions (PBS) (1mg/mL) with different temperature (20 and 37 °C) and transferred into dialysis bags with a molecular weight cut off of 3500. Dialysis bags were then put into 30 mL PBS solutions for release. At given time intervals, 4 mL of aliquot was taken out to measure the DOX concentration in the dialysate with a UV–VIS spectrophotometer. Additionally, 4 mL of the corresponding fresh PBS solution was added after each sampling to ensure that the total volume of the buffer solution remained constant. The cumulative release was calculated by using Equation (2) as follows:

(1) $$\text{Cumulative release }(\%)=\frac{100\times \left(30.0C_{n}+4.0\sum C_{n-1}\right)}{W_{0}}$$

where *W*_0_ (mg) is weight of drug in the polymer; *C_n_* (mg/mL) is the concentration of DOX in buffer solution, which was withdrawn for *n* times, *C_n−_*_1_ (mg/mL) is the concentration of DOX in buffer solution, which was withdrawn for *n* − 1 times.

## 4. Results and Discussion

**Figure 1:**
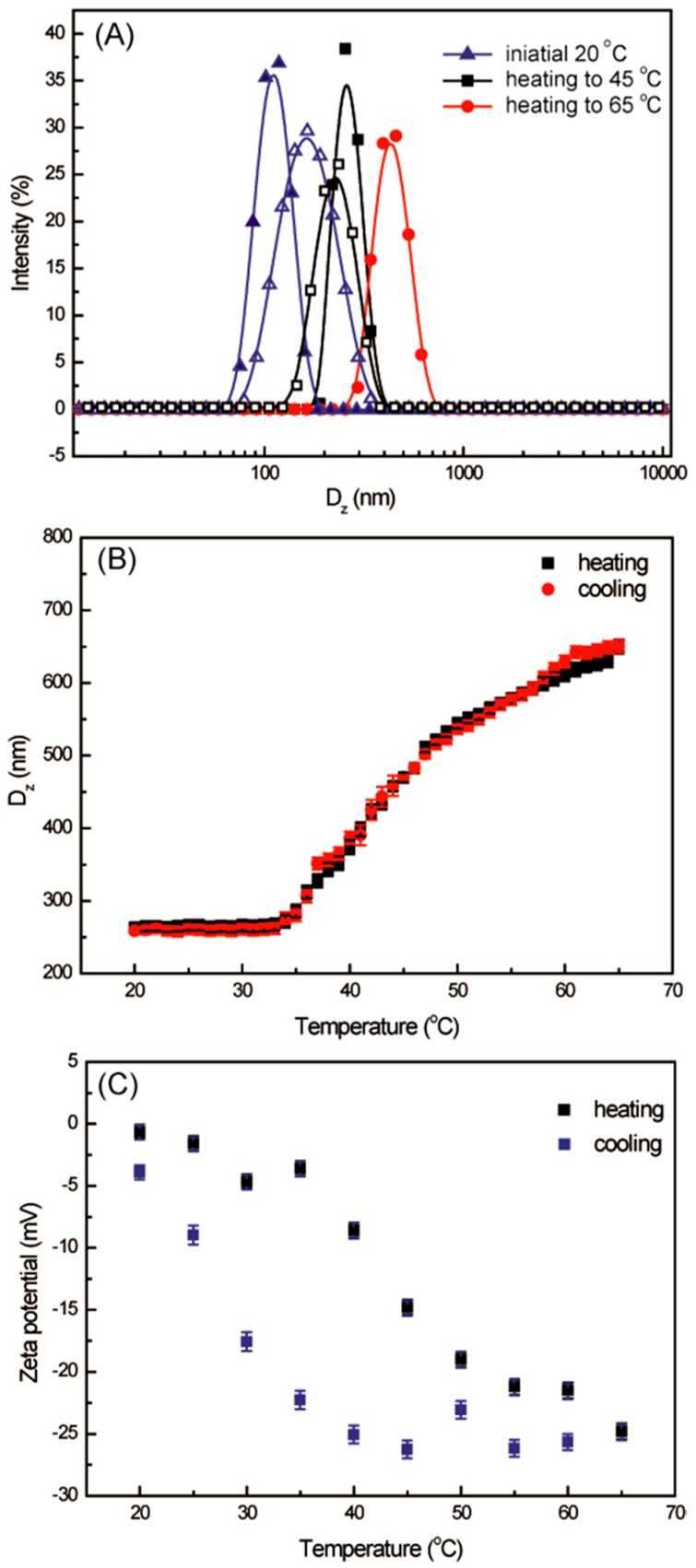
Z-average diameter distributions (**A**); Z-average diameter values depend on temperature (**B**) and Zeta-potential (**C**) of LCHBPNIPAM self-assemblies in aqueous solution (0.2 mg·mL^−1^) heating from 20 to 65 °C (solid lines), and then cooling to 20 °C (dash lines).

The resulting polymer LCHBPNIPAM (*M*_n,SEC-MALLS_ = 44,300 Da, *M*_w_/*M*_n_ = 1.27; η_n_ = 7.0, α = 0.43) was obtained by the click chemistry of the AB_2_ macromonomer (*M*_n,SEC-MALLS_ = 4200 Da, *M*_w_/*M*_n_ = 1.20) according to our previously work [38]. Generally, the backbone-thermoresponsive hyperbranched polymer based on hydrophilic-hydrophobic balance can self-assemble to form multi-compartment vesicles below lower critical solution temperature (LCST), and the micelles aggregate to larger particles above LCST [39,43,44,45,46,47]. Thus, the thermo-induced reversible self-assembly behavior of LCHBPNIPAM with an intensely increasing Z-average diameter (*D_z_*) values at 35 °C (LCST point) (Figure 1B) was easily promoted via directly dissolving it in water with a concentration of 0.2 mg/mL at a cyclic temperature of heating from 20 to 65 °C, and then cooling to 20 °C. Dynamic/static light scattering (DLS/SLS), transmission electron microscopy (TEM), fluorescence spectrophotometry (FS), and ^1^H NMR (in D_2_O) measurements were conducted to obtain deeper insight into the self-assembly morphology and size.

DLS was first used to study the size of LCHBPNIPAM in aqueous solution as shown in Figure 1A,B. It was found that aggregates with a Z-average diameter (*D_Z_*) ranging from 264 to 637 nm were formed as the temperature was raised from 20 to 65 °C, revealing the occurrence of thermally-induced self-assembly [48]. However, the *D_Z_* value came back to 258 nm as the solution cooling to 20 °C. On the other hand, the change of zeta-potential of LCHBPNIPAM self-assemblies also reflected the reversibility of the self-assembly behaviors (Figure 1C and Table 1). It was notable that the zeta potential values had a “transition temperature” (*ca.* 35 °C) corresponding to the size transition. The absolute values of Zeta-potential showed an drastic decreasing from −0.64 to −23.2 mV, and then reducing to −3.58 mV, corresponding to the temperature increasing from 20 to 65 °C and decreasing to 20 °C, respectively. The Figure 1C shows that the ionization of secondary amine group of PNIPAM chains induced a negative charge in the self-assemblies surface at room temperature. However, the graph clearly demonstrated that the further ionization of the secondary amine group of PNIPAM causes a significant decrease in zeta potential at temperatures above the LCST. The above change tendency was in accordance with the results of size of aggregates [49,50,51,52]. One possible reason for the change of zeta potential values *vs.* temperature is that the ionization of secondary amine group of PNIPAM chains is an endothermic process, so increasing the solution temperature may enhance the ionization behavior, further leading to the change of zeta potential as temperature. Additionally, increasing the temperature can promote the coalescence of self-assemblies, and further increase the surface negative charge density of self-assemblies. To further confirm the reversibility of the self-assembly process, we utilized a combination of SLS and DLS techniques to discriminate the inner structure of LCHBPNIPAM self-assemblies at initial and final states, respectively. It is known that the *R_g_*/*R_h_* value can predict the particle morphology [40]. For example, a solid sphere has an *R_g_*/*R_h_* value of 0.774, while a thin-layer hollow sphere of 1.00. The ratio *R_g_*/*R_h_* is useful for analyzing the structure of a nano-sized particle, since ratios close to 1.0 indicate that a hollow particle has been formed [40,53]. As shown in Table 1, the *R_g_*/*R_h_* values of LCHBPNIPAM self-assemblies were 0.96 and 1.07 at initial and final states, respectively. Therefore, the *R_g_*/*R_h_* values 0.96 and 1.07 indicated a vesicular structure with a hollow cavity. The *R_g_*/*R_h_* ratio for the micelles is equal to 1.0. This supports the TEM observation of hollow characteristics. Furthermore, the results of SLS confirmed the reversibility of self-assembly process and the formation of multi-compartment vesicles.

**Table 1:** Physicochemical parameters of LCHBPNIPAM self-assemblies in aqueous solution (0.2 mg·mL^−1^).

Temperature (°C)	*D_av,TEM_* (nm) ^a^	*D_z_* (nm) ^b^	PDI ^c^	Zeta (mV) ^d^	*R_g_*/*R_h_* ^e^
20	194 ± 0.6	264 ± 0.4	0.504	−0.6 ± 0.3	0.96
45 heating	345 ± 0.4	469 ± 0.5	0.248	−14.8 ± 0.4	-
65 heating	540 ± 0.4	637 ± 0.3	-	−23.2 ± 1.2	-
45 cooling	301 ± 0.5	471 ± 0.4	0.183	−26.2 ± 0.7	-
20 cooling	222 ± 0.6	258 ± 0.6	0.275	−3.6 ± 0.5	1.07

^a^ Average diameter determined by TEM; ^b^ Z-Average diameter determined by DLS (Repeating three times); ^c^ Polydispersity determined by DLS; ^d^ Zeta-potential determined by DLS; ^e^ Radius of gyration (*R_g_*) determined by SLS and hydrodynamic radius (*R_h_*) determined by DLS.

**Figure 2:**
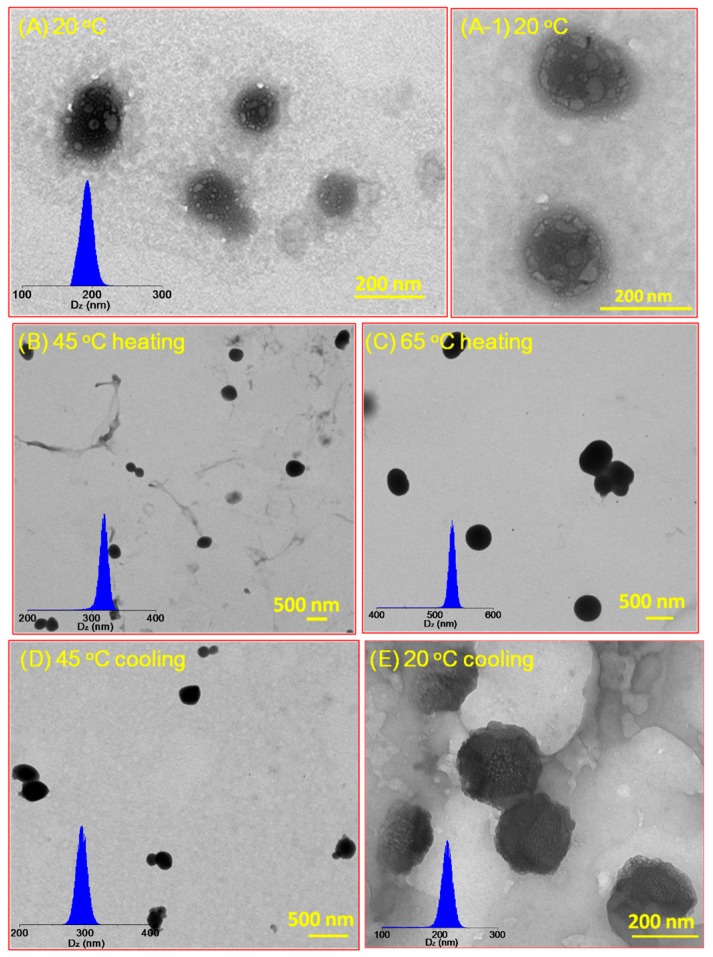
Typical TEM images obtained by drying aqueous solutions of LCHBPNIPAM self-assemblies (0.2 mg/mL) at 20 °C (**A**); 45 °C (heating) (**B**); 65 °C (heating) (**C**); 45 °C (cooling) (**D**); and 20 °C (cooling) (**E**).

**Figure 3:**
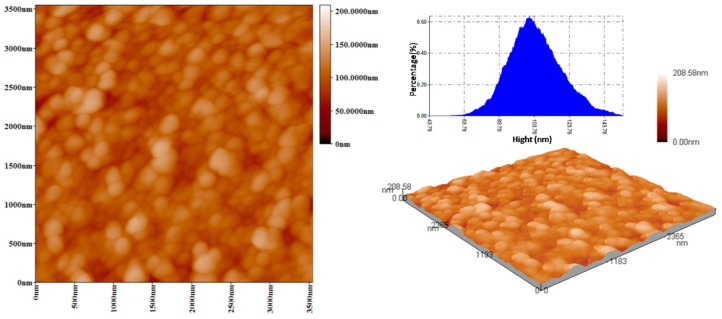
Typical AFM images and its height analysis of LCHBPNIPAM self-assemblies (0.2 mg/mL) at 20 °C.

TEM was employed to further demonstrate the size and morphology of LCHBPNIPAM self-assemblies in aqueous solution at different temperatures, as shown in Figure 2. With a freeze-drying process, the intermediate morphologies of the aggregates can be well preserved in this method [39,41,42,54]. As can be noticed in Figure 2A–E, the morphologies of self-assemblies changed from multi-compartment vesicles to solid micelles and to multi-compartment vesicles again in accordance with increasing the temperature from 20 to 65 °C and then decreasing to 20 °C. Being estimated from TEM images, the multi-compartment vesicles had an average diameter (*D_av_*) of 194 nm at 20 °C (Figure 2A,(A-1)). Gradually, heating to 45 °C led to an increase of *D_av_* value to 345 nm (Figure 2B) due to the phase transitions of PNIPAM segments. Moreover, the size of self-assemblies grew to 540 nm as the temperature increasing to 65 °C (Figure 2C). In order to prove the reversible self-assembly, we made the aqueous solution cooling to 45 and 20 °C, accompanied with the gradually decreasing size of self-assemblies from 540 to 301 nm at 45 °C, and then 222 nm at 20 °C (Figure 2D,E). Moreover, AFM images of the multi-compartment vesicles at 20 °C (Figure 3) was obtained by tapping-mode AFM for the specimen cast on a mica substrate. Such multi-compartment vesicles confirmed a width of 186 ± 1.3 nm (Figure 3), consistent with the width measured by TEM. The layer height was determined to be 103 ± 3 nm (Figure 1B). The above change of size of self-assemblies was in accordance with the results of DLS experiments as shown in Table 1.

**Figure 4:**
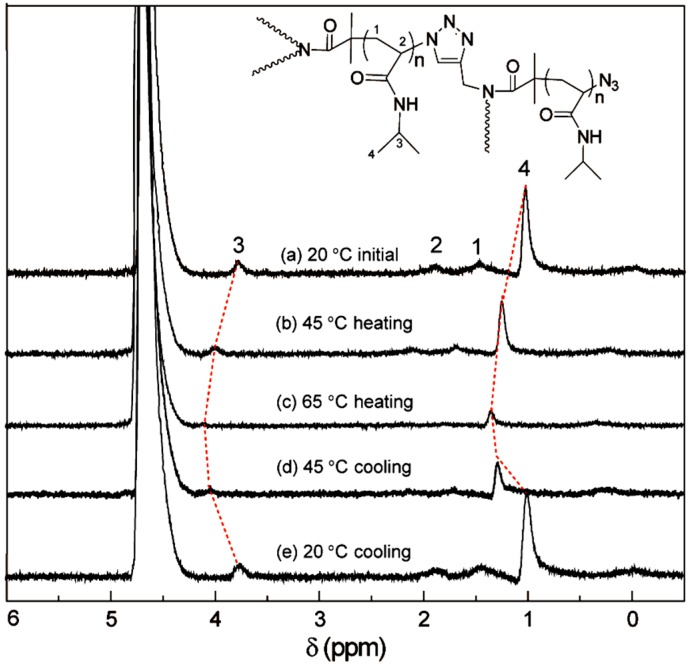
^1^H NMR spectra of LCHBPNIPAM self-assemblies in D_2_O at (**a**) 20 °C; (**b**) 45 °C (heating); (**c**) 60 °C (heating); (**d**) 45 °C (cooling); (**e**) 20 °C (cooling).

We also explored the inner architecture of LCHBPNIPAM self-assemblies by ^1^H NMR in D_2_O under different temperatures (Figure 4). It was obvious that all signals of PNIAPM segments were visible at 20 °C (Figure 4a). However, the proton peaks were weaken with a slight downfield shift after increasing the temperature to 45 °C (Figure 4b). Moreover, most proton peaks disappeared at 65 °C, revealing that PNIPAM segments collapsed as hydrophobic core layer of LCHBPNIPAM self-assemblies. Furthermore, the signals of PNIPAM chains appeared again along with the solution temperature cooling from 65 to 45 and 20 °C (Figure 4d,e). The above results indicated that the self-assembly process of LCHBPNIPAM in aqueous solution was reversible based on the thermoresponsive long-chain PNIPAM backbone.

**Figure 5:**
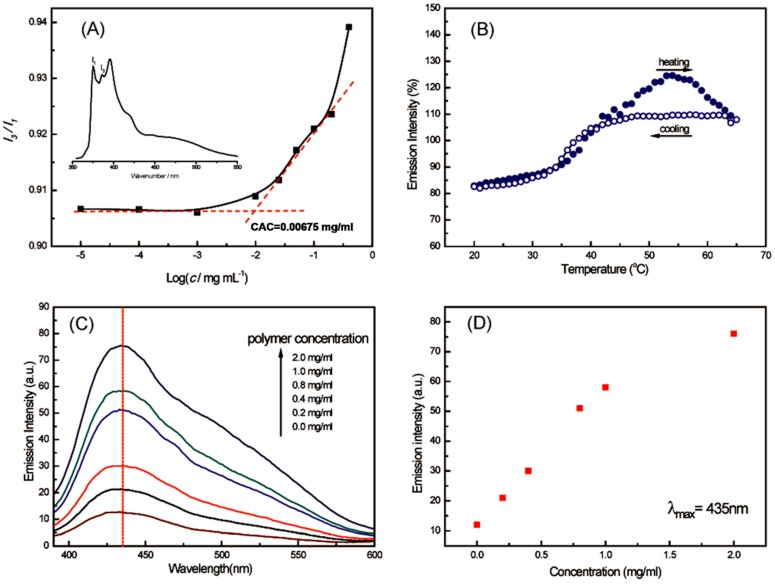
(**A**) Relationship between the fluorescence intensity ratio (*I*_3_/*I*_1_) and LCHBPNIPAM concentration in aqueous solution (Inset: fluorescence emission spectrum of pyrene); (**B**) maximum values of ANS aqueous solution (0.05 mM, λ_max_ = 435 nm) in the presence of LCHBPNIPAM solution (0.2 mg/mL) with the gradual elevation and reduction of temperature; (**C**) fluorescence spectra of ANS in the presence of LCHBPNIPAM with various concentrations at 20 °C; and (**D**) maximum values of ANS emission intensity at various LCHBPNIPAM concentrations ((ANS) = 0.05 mM).

FS spectroscopy was employed to further confirm the formation, reversibility and encapsulation functionality of LCHBPNIPAM self-assemblies. The critical aggregation concentration (CAC), which acted as a key parameter to quantitatively confirmation whether the micelles had been formed [55], was estimated by FS using pyrene as a hydrophobic probe. The ratio of the intensity of the third and first peaks (*I*_3_/*I*_1_) in the emission spectrum was very sensitive to the polarity of the medium surrounding pyrene molecules [56]. The CAC was obtained from the intersection of the baseline and the tangent of the rapidly rising *I*_3_/*I*_1_ curves, indicating the formation of self-assemblies. Thus, the *I*_3_/*I*_1_ value of the pyrene emission spectra *versus* the logarithm of the LCHBPNIPAM concentration was 0.00675 mg/mL, as shown in Figure 5A. Furthermore, 8-Anilino-1-naphthalenesulfonic acid ammonium salt hydrate (ANS) was selected as the guest molecule because its main absorption peak can be easily detected by FS spectroscopy in an aqueous solution [57,58]. Emission spectra of an ANS-containing LCHBPNIPAM solution with a concentration of 0.2 mg/mL was recorded as the solution heating from 20 to 65 °C and then cooling to 20 °C. As shown in Figure 5B, a sudden increasing of fluorescence intensity at λ_max_ = 435 nm from 35 to 55 °C indicated that the releasing of the 1,8-ANS molecules into aqueous surroundings accompanied with the thermal collapsing of PNIPAM segments [58]. The intensity of ANS-containing solution became the same as the intensity at origin state when the temperature came back to 20 °C, indicating that the micelles came back to original state. Furthermore, the encapsulation function of the formed LCHBPNIPAM self-assemblies was also proved by the FS technique. Figure 5C presented the fluorescence spectra of ANS in the presence of LCHBPNIPAM with various concentrations at 20 °C. It was found that the peak intensities of ANS in LCHBPNIPAM solutions regularly increased with the increase of the LCHBPNIPAM concentration. Figure 5D showed that the emission intensities at the λ_max_ value of ANS increased with increasing LCHBPNIPAM concentration. The above results indicated that ANS guests were encapsulated into LCHBPNIPAM self-assemblies according to our previous work [57,59].

These above-mentioned results suggested that the thermo-induced reversible self-assembly of LCHBPNIPAM did, indeed, occur. Herein, the possible self-assembly mechanism was proposed. The hydrophilic PNIPAM segments form multi-compartment vesicles, membrane, and coronas, respectively [60], while the hydrophobic terminal alkynyl groups are located in the center of the multi-compartment vesicle membrane at 20 °C (Scheme 1a,b). Furthermore, PNIPAM segments collapsed to form solid core above LCST (Scheme 1b,c). On the contrary, the multi-compartment vesicles membrane and coronas are formed by PNIPAM segments again corresponding with cooling to 20 °C (Scheme 1b,c). In our opinion, active hydrogen existing in the unsaturated triple bonding of the alkynyl group and the amido group of PNIPAM may form intermolecular hydrogen bonding with an oxygen atom and the emergence of hydrogen bonds CONH···CON−, leading to the formation of different structural self-assemblies.

**Figure 6:**
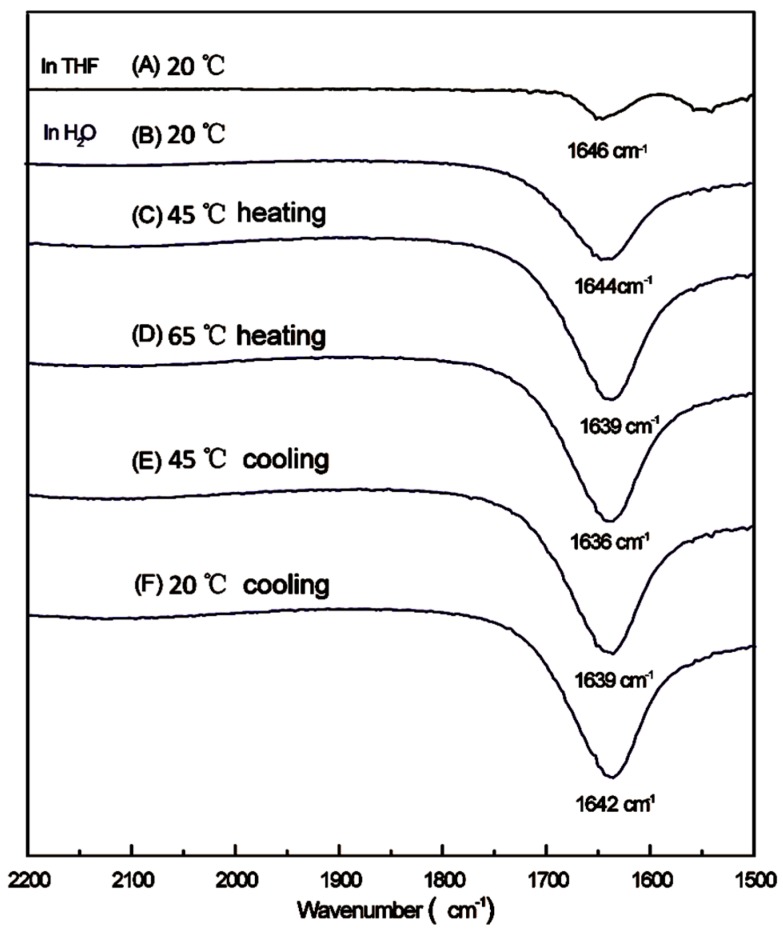
ATR-FTIR spectra of LCHBPNIPAM in THF (**A**) and in H_2_O at different temperatures (**B**–**F**) ((**B**) 20 °C; (**C**) 45 °C (heating); (**D**) 60 °C (heating); (**E**) 45 °C (cooling); and (**F**) 20 °C (cooling)).

In order to confirm the existence of hydrogen bonding in the self-assembly process, ATR-FTIR was used in this work. As shown in Figure 6, the ATR-FTIR spectra of LCHBPNIPAM in THF and in H_2_O under different temperatures were compared, one significant change could be seen in the absorbance peak of the C=O groups. The peak at about 1646 cm^−1^ in THF (Figure 6A), shifted to a lower wavenumber of 1644 cm^−1^ in H_2_O (Figure 6B) at 20 °C, along with the expanding and strengthening of C=O absorbance. What is more, the peak further shifted to the wavenumber of 1636 cm^−1^ with the temperature increasing to 65 °C (Figure 6B–D). The formation of strong inter/intra-polymer hydrogen bonding might contribute to the above obvious shifting in the LCHBPNIPAM spectra. This result was in agreement with a report on the hydrogen bonding-mediated vesicular self-assembly by Du *et al.* [53]. It should be noted that the peaks of C=O absorbance came back to 1644 cm^−1^ as the solution cooling to 20 °C (Figure 6E,F). Therefore, the inter/intra-polymer hydrogen bonding, indeed, existed in LCHBPNIPAM self-assemblies.

**Figure 7:**
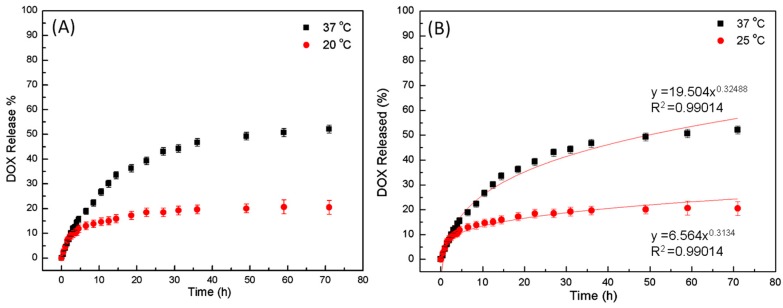
(**A**) Release profiles of DOX from LCHBPNIPAM self-assemblies as a function of time at 20 °C (red line) and 37 °C (black line); and (**B**) the profile of drug release mechanism based on the Higuchi kinetics and the Korsmeyer–Peppas semi-empirical equation under 20 and 37 °C.

The different LCHBPNIPAM self-assemblies, including multi-compartment vesicles (below LCST) and solid micelles (above LCST) could be utilized as a feasible medium for encapsulation and release of drug molecules (Scheme 1). Thus, DOX as a model drug was loaded into the multi-compartment vesicles or micelles for the release experiments. Measurements of DOX release provided a quantitative result of the morphology-controlled DOX release. The release curves of DOX were investigated under different temperature in the solution as shown in Figure 7A. In case of release from system loaded with DOX the release rate at 37 °C was higher than that of 20 °C, and the maximum amount of release drug is 20% and 50% for 20 and 37 °C, respectively. Furthermore, the release mechanism of DOX was analyzed with the Higuchi kinetics and the Korsmeyer–Peppas semi-empirical equation (Equation (1)) [61,62,63]. In the equations, *M_t_*/*M_∞_* is the fraction of the drug released at time *t*, *K* is a kinetic constant incorporating structural and geometric characteristics of the device, and n is the release exponent, indicative of the mechanism of drug release. The *n* value of the Korsmeyer–Peppas model plot under 20 °C was 0.313 with an *R*^2^ value of 0.939 and under 37 °C was 0.325 with an *R*^2^ value of 0.990 (Figure 7B). This result validated that the release processes were consistent with Fickian diffusion kinetics [60]. The temperature-dependent release of DOX was attributed to the thermosensitive property of PNIPAM segments. As discussed above, the multi-compartment vesicles inhibited the release of DOX at 20 °C. While PNIPAM segments collapsed to form solid core above LCST may result in some crack in the micelles. This could further accelerate the release of encapsulated DOX. Therefore, the DOX release from the loaded self-assemblies further proved the morphology transitions of LCHBPNIPAM self-assemblies below and above LCST.

(2) $$\frac{M_{t}}{M_{\infty }}=Kt^{n}$$

## 5. Conclusions

In conclusion, the introduction of thermoresponsive poly(*N*-isopropyl acrylamide) segments onto long chain hyperbranched polymer (LCHBPNIPAM) backbone can induce a reversible self-assembly. During the heating-cooling process, the morphology of LCHBPNIPAM self-assemblies changed from multi-compartment vesicles to solid micelles and back to multi-compartment vesicles again, and the size underwent a first increase and then decrease. The intermolecular hydrogen bonding plays an important role in forming various self-assembly structures of LCHBPNIPAM at different temperatures. The release rate of Doxorubicin from different LCHBPNIPAM self-assemblies can be effectively controlled. Therefore, these research results may be helpful to extend the application of long-chain hyperbranched polymers in drug delivery system.
